# An Integrated Strategy Based on 10-DAB Extraction and In Situ Whole-Cell Biotransformation of Renewable *Taxus* Needles to Produce Baccatin III

**DOI:** 10.3390/molecules29112586

**Published:** 2024-05-31

**Authors:** Ping Kou, Yingying Yu, He Wang, Yuchi Zhang, Zhaoxia Jin, Fang Yu

**Affiliations:** School of Biological Engineering, Dalian Polytechnic University, Dalian 116034, China

**Keywords:** baccatin Ⅲ, whole-cell biotransformation, *Taxus* needles, 10-deacetylbaccatin III-10-β-O-acetyltransferase, acetyl-CoA supply

## Abstract

Baccatin III is a crucial precursor in the biosynthesis pathway of paclitaxel. Its main sources are extraction from *Taxus* or chemical synthesis using 10-deacetylbaccatin III (10-DAB) as substrate. However, these preparation approaches exhibit serious limitations, including the low content of baccatin III in *Taxus* and the complicated steps of chemical synthesis. Heterologous expression of 10-deacetylbaccatin III-10-O-acetyltransferase (TcDBAT) in microbial strains for biotransformation of 10-DAB is a promising alternative strategy for baccatin III production. Here, the promotion effects of glycerol supply and slightly acidic conditions with a low-temperature on the catalysis of recombinant *TcDBAT* strain were clarified using 10-DAB as substrate. *Taxus* needles is renewable and the content of 10-DAB is relatively high, it can be used as an effective source of the catalytic substrate 10-DAB. Baccatin III was synthesized by integrating the extraction of 10-DAB from renewable *Taxus* needles and in situ whole-cell catalysis in this study. 40 g/L needles were converted into 20.66 mg/L baccatin III by optimizing and establishing a whole-cell catalytic bioprocess. The method used in this study can shorten the production process of *Taxus* extraction for baccatin III synthesis and provide a reliable strategy for the efficient production of baccatin III by recombinant strains and the improvement of resource utilization rate of *Taxus* needles.

## 1. Introduction

Paclitaxel is recognized worldwide as one of the most effective cancer chemotherapeutic agents, and has been widely used in the clinical treatment of breast cancer, lung cancer and colorectal cancers [1,2,3]. Its supply mainly relies on the chemical semi-synthetic method which uses the more abundant precursor compound 10-deacetylbaccatin III (10-DAB) in *Taxus* species as substrate. Then, 10-DAB is converted into paclitaxel using a multi-step chemical synthesis [4,5]. The first key step is to produce baccatin Ⅲ through the C10-hydroxyl acetylation of 10-DAB [6]. This acetylation process has been extensively optimized but still requires at least three steps in the presence of catalyst. Furthermore, some toxic chemicals are usually applied under harsh reaction condition, such as acetic anhydride and triethylsilane [7,8]. Therefore, developing an eco-friendly, efficient and mild transformation method is promising for the preparation of baccatin Ⅲ.

Compared with the conventional chemical methods, the enzyme conversion from 10-DAB to baccatin Ⅲ has shown many superiorities, such as a stronger selectivity, higher efficiency and more eco-friendly [9,10,11]. Currently, research on enzymes that can catalyze the C10-hydroxyl acetylation has focused on the 10-deacetylbaccatin III-10-β-O-acetyltransferase (DBAT) enzymes from the *Taxus* plant, with the exception of a few enzymes derived from microorganisms. DBAT can directly catalyze the acetylation from 10-DAB to baccatin Ⅲ in one step when acetyl-CoA is the acetyl donor in the biosynthesis pathway of paclitaxel in *Taxus* plants [12,13]. However, the slow growth of *Taxus* plant and the low expression of DBAT encoding genes limit the application potential of baccatin Ⅲ production from plant materials. Therefore, the rapid production of baccatin Ⅲ is expected to be achieved by expressing DBAT in rapidly growing microorganisms in the presence of the substrate 10-DAB.

In particular, the acetylation process of 10-DAB to baccatin III requires acetyl-CoA as an acetyl donor. The addition of acetyl-CoA after heterologous expression of DBAT would make the application more costly. Moreover, most of the commonly used biotransformation methods require multi-step isolation and purification to obtain the target enzyme, which improves the catalytic efficiency but makes the process steps more cumbersome [14,15]. Therefore, using *Escherichia coli* (*E. coli*) whole cells as catalysts instead of the purified enzyme to catalyze the target reaction not only ensures the supply of acetyl groups through its own metabolism pathway, but also omits the process of enzyme purification [16,17].

The production of baccatin Ⅲ requires a supply of the substrate 10-DAB in addition to the presence of acetyl-CoA as described above. Currently, the main source of 10-DAB is extracted from the *Taxus* plant. The use of high-purity 10-DAB as common substrate for the production of baccatin III requires a series of impurity removal and enrichment operations of *Taxus* extract [18]. The *Taxus* plant grows slowly and has been over-harvested [19,20,21]. If the bark and stem are used to provide the substrate 10-DAB, plant growth and development will be significantly affected, which may result in the reduction of *Taxus* resources. The needle is a suitable plant source of 10-DAB because of its significant advantages of higher 10-DAB accumulation, renewability and large resource reserves [22,23]. *Taxus chinensis* is the most widely distributed *Taxus* species in China. The content of 10-DAB is relatively high, which is about 0.50–3.00 mg/g [24,25]. The needles can be harvested after three years of artificial cultivation. Therefore, directly using *Taxus chinensis* needles as a supply source of 10-DAB for baccatin Ⅲ production is an effective strategy to improve the utilization efficiency and value of *Taxus* needles resources.

In this study, the *TcDBAT* gene from *Taxus chinensis* was heterologous expressed in *E. coli*. The 10-DAB was used as the substrate to investigate the effects of key factors on baccatin III production during whole-cell biotransformation process. The patterns for the effects of pH, carbon source and temperature on the biotransformation process were comprehensively analyzed. The most suitable whole-cell biotransformation condition was optimized and determined by using readily-available and renewable *Taxus chinensis* needles as substrate. In this study, an integrated bioprocess of extracting 10-DAB from *Taxus* needles and converting it into baccatin III in situ was established, which provide a reference strategy for the high-value utilization of the *Taxus* needles and the efficient production of baccatin III.

## 2. Results

### 2.1. Heterologous Expression of TcDBAT and Condition Optimization

**Figure 1:**
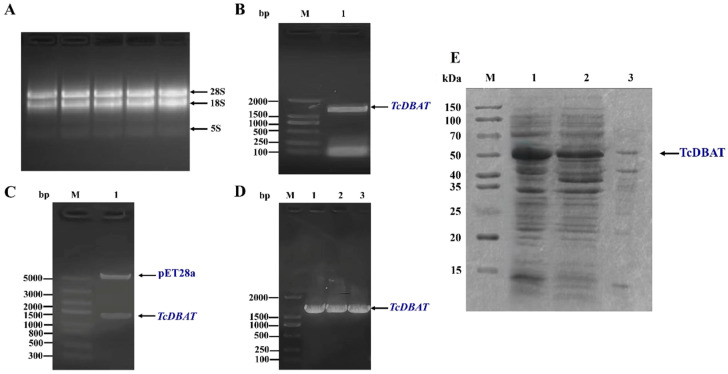
Cloning and heterologous expression of *TcDBAT*. (**A**) Total RNA of *Taxus chinensis* needles. (**B**) PCR amplification of *TcDBAT*. (**C**) Identification of pET28a-*TcDBAT* by double enzyme digestion. (**D**) Colony PCR identification of pET28a-*TcDBAT*. (**E**) SDS-PAGE analysis of recombinant TcDBAT after IPTG induction expression. M: marker; 1: whole cell; 2: supernatant after cell disruption; 3: precipitate after cell disruption.

The biosynthesis of baccatin III was mainly achieved in *Taxus* plants. Therefore, the target acetyltransferase gene was amplified from *Taxus chinensis* by a specific primer and the target fragment showed 99% similarity to *TcDBAT* (GenBank accession number: AY365031.2) using colony PCR, enzyme digestion and sequencing verification (Figure 1A–D). Then, *TcDBAT* was expressed in *E. coli* using the pET-28a expression vector. The SDS-PAGE analysis showed that the target protein located at a protein size of about 49 kDa, which indicated the successful expression of TcDBAT in *E. coli* (Figure 1E). The majority of TcDBAT was expressed as soluble proteins with a very small fraction of proteins in inclusion bodies.

The whole fermentation process of the whole-cell catalysis begins with the induced expression of *TcDBAT* in *E. coli* and the main factors affecting this process are the timing of induction (cell density) and the concentration of the inducer (IPTG). Therefore, in order to explore the influence of induction timing and IPTG concentration on the target enzyme activity, 10-DAB standard was used as substrate to determine the catalytic efficiency of TcDBAT under different *E. coli* cell densities and IPTG concentrations. Figure S1A shows that the conversion rate of 10-DAB and baccatin III yield increased and then decreased with the continuous increase in *E. coli* density. The conversion rate and yield was the highest at OD_600_ = 0.6 and reached 5.33-fold and 7.78-fold of that at OD_600_ = 1, respectively. Figure S1B exhibits that the conversion rate and the yield also increased and then decreased with the increasing concentration of IPTG. The highest conversion and yield were achieved at 0.1 mM, which were 3.78 and 3.59 times higher than that of 0.2 mM, respectively. The above results indicated that the catalytic efficiency of TcDBAT expressed in *E. coli* was significantly affected by the timing of induction and the concentration of IPTG under the ideal mode of high concentration of substrate. Meanwhile, OD_600_ = 0.6 and 0.1 mM IPTG were identified as the most suitable conditions for inducing the expression of TcDBAT.

### 2.2. Thermostability during Biotransformation of 10-DAB

**Figure 2:**
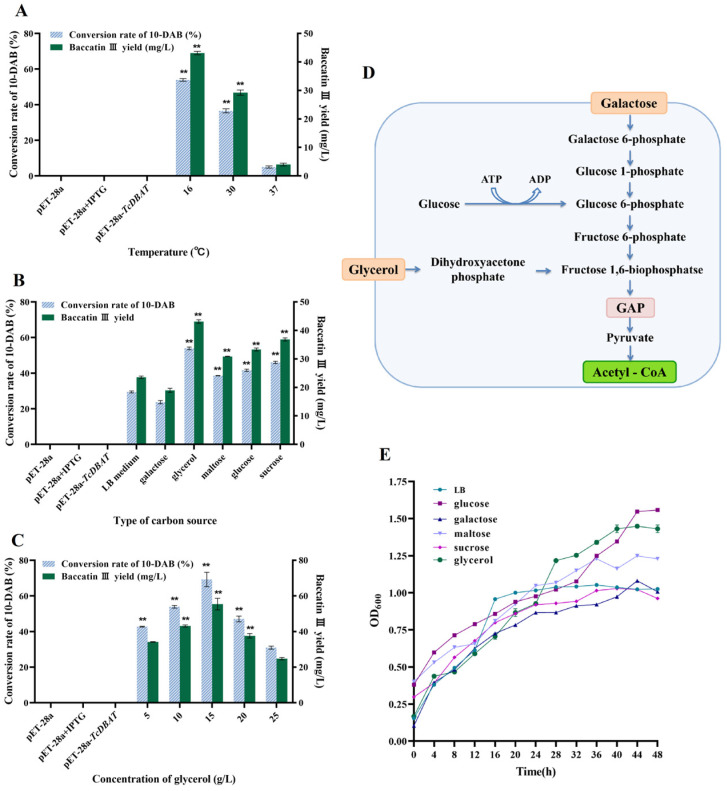
Effect of temperature and carbon source on the whole-cell catalytic synthesis of baccatin III. (**A**) Effect of temperature on the whole-cell catalytic. (**B**) Effect of carbon source type on the whole-cell catalytic. (**C**) Effect of carbon source concentration on the whole-cell catalytic. (**D**) Metabolic pathway for the synthesis of acetyl-CoA from representative carbon sources. (**E**) Effect of different carbon sources on strain growth. ** represents significant differences at the *p* < 0.01 level.

Although TcDBAT is highly specific, these plant-derived genes generally have low thermostability [26,27]. Therefore, exploring the effect of fermentation temperature on catalytic efficiency and determining the appropriate catalytic temperature can guide the efficient synthesis of baccatin III in the subsequent process. Figure 2A shows the catalytic capacity of TcDBAT at common fermentation temperatures (16 °C, 30 °C and 37 °C). Baccatin III can hardly be detected when catalyzed at 37 °C, indicating that the target biotransformation process cannot occurred successfully at 37 °C. This result may be determined by the structure and thermal sensitivity of TcDBAT. The yield of baccatin III reached 43.12 mg/L and 29.23 mg/L at 16 °C and 30 °C, respectively. These results indicated that a relatively low temperature is more conducive to the catalytic activity of TcDBAT, which is basically consistent with previous studies.

### 2.3. Adjustment of Medium Carbon Source to Promote 10-DAB Biotransformation

Acetyl-CoA is the acetyl donor during the biotransformation process of 10-DAB and can directly affect the production of baccatin III. Therefore, ensuring the adequate supply of acetyl-CoA is crucial for the smooth occurrence of the target catalytic process. Acetyl-CoA is a central metabolite in cell growth and metabolism, and different carbon sources can produce acetyl-CoA through different metabolic pathways [28,29]. Therefore, investigating the effect of carbon sources on baccatin III production can further clarify the relationship between the metabolic supply of acetyl-CoA and the target biotransformation process. Six carbon sources commonly used in microbial fermentation processes were selected for exploring the effect of the carbon source on baccatin III production. Figure 2B shows that the conversion of 10-DAB and the yield of baccatin III were highest when glycerol was used as the carbon source, which were 17.01–127.18% higher than LB medium, galactose, maltose, glucose and sucrose. The reason for this may be that the pathway of acetyl-CoA production from glycerol metabolism can be completed in only four steps, which can release the excess acetyl-CoA from the metabolic equilibrium and act as an acetyl donor to ensure the target acetylation process to proceed smoothly [30]. It is worth noting that the most significant increase in the yield of baccatin III (127.18%) was observed when galactose was used as the carbon source. Therefore, taking galactose as an example, it can be found that its route for acetyl-CoA production requires eight steps, including the biosynthesis of glucose-1-phosphate and fructose-6-phosphate, which is longer and more complicated than glycerol metabolism (Figure 2D). This may lead to a low synthesis efficiency of acetyl-CoA and make it difficult to meet the reaction requirements of the target acetylation process, resulting in a lower yield of baccatin III.

In addition, the cell density can reflect the growth status of the engineered strain. Figure 2E reveals that the cell density of different carbon sources gradually increased with time and the type of carbon source affected the growth status. The highest cell density was found when glycerol and glucose were the carbon sources, which indicated the most active growth of the strain. This result was consistent with the highest yield of baccatin III when glycerin was selected. This may be due to the strong supply of acetyl-CoA by individual cells, or simply due to the increase in cell number. The cell density was lowest when sucrose was used as carbon source, but not galactose. These results suggest that cell density is not the most critical factor affecting acetylation and it can be inferred that the efficiency of the biotransformation process is primarily determined by the ability of individual cells to supply acetyl-coA under different carbon sources.

The concentration of the carbon source also affects the catalytic process and growth status, so a gradient of glycerol concentration was chosen for exploring its effect on the biotransformation of 10-DAB. Figure 2C shows that the conversion of 10-DAB III and yield of baccatin III increased and then decreased with concentration of glycerol. The highest yield of baccatin III (55.40 mg/L) was achieved at a glycerol concentration of 15 g/L. The reason for this may be that as the amount of carbon source increased, the nutrient requirements of strain and the demand for acetyl-CoA supply for the biotransformation reached saturation. The continuous increase in the carbon source can not further promote the target acetylation process, but may inhibit the synthesis of baccatin III by changing the composition of the fermentation broth.

### 2.4. Slightly Acidic Condition Conducive to the Biotransformation of 10-DAB to Baccatin III

**Figure 3:**
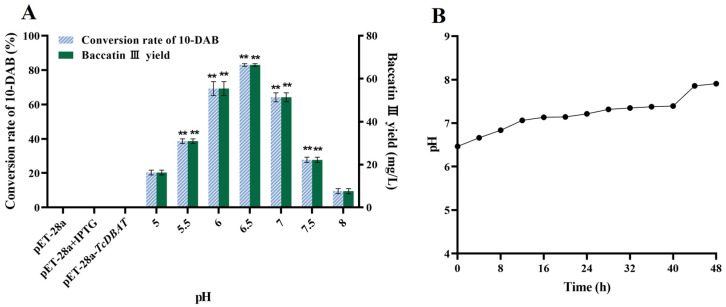
Effect of pH on the whole-cell catalytic process of baccatin III. (**A**) Effect of pH on the whole-cell catalytic synthesis of baccatin III. (**B**) Change of pH during biotransformation (initial pH = 6.5). ** represents significant differences at the *p* < 0.01 level.

The pH condition is also a factor affecting the target acetylation process. Therefore, exploring the effect of pH on the target catalytic process can guide subsequent applications for baccatin III synthesis by biotransformation. The conversion rate of 10-DAB and the yield of baccatin III gradually increased and then decreased with the initial pH from acidic to basic (Figure 3A). The highest conversion of 10-DAB (45.28%) and the yield of baccatin III (66.40 mg/L) were achieved at a pH of 6.5, respectively. This indicated that the target whole-cell catalytic process can be most preferably achieved in slightly acidic condition. The overly acidic or alkaline environment may slow down the target catalytic process by inhibiting microbial growth or inhibiting the forward conversion of 10-DAB to baccatin III.

Then, a pH of 6.5 was chosen as an example. The patterns of pH variation during the whole catalytic process were investigated to further clarify the mechanism of its effect on biotransformation of 10-DAB. As shown in Figure 3B, the pH gradually increased with the extension of time. This is probably because of the continuous metabolism of carbon source and the production of other metabolites. In particular, some studies have found that alkaline conditions inhibited the conversion of 10-DAB to baccatin III; instead, these conditions promoted the reversible reaction of baccatin III to 10-DAB [31]. Therefore, a high initial pH would directly lead to a decrease in the yield of baccatin III due to the reversible reaction. This view is consistent with the results of the present study. On the contrary, a low initial pH was beneficial for the target acetylation process but led to a decrease in the baccatin III yield by inhibiting the growth of microorganisms. In brief, the choice of initial pH should balance the relationship between microbial growth and the forward progress of the target catalytic reaction.

### 2.5. Establishment of Process for the Production of Baccatin III by Whole-Cell Biotransformation Based on Taxus Needles

The influence pattern of multiple factors on the target biotransformation process have been clarified by adding 10-DAB as the substrate. However, the main source of 10-DAB was extracted and purified from the *Taxus* resource with cumbersome isolation steps. Therefore, the simultaneous extraction of 10-DAB from *Taxus* needles and the synthesis of baccatin III with an in -situ biotransformation can shorten the production process. The establishment of the optimal process for the production of baccatin III using whole-cell biotransformation with *Taxus chinensis* needles can provide a novel strategy for the high-value utilization of *Taxus* needles resources and the production of baccatin III.

**Figure 4:**
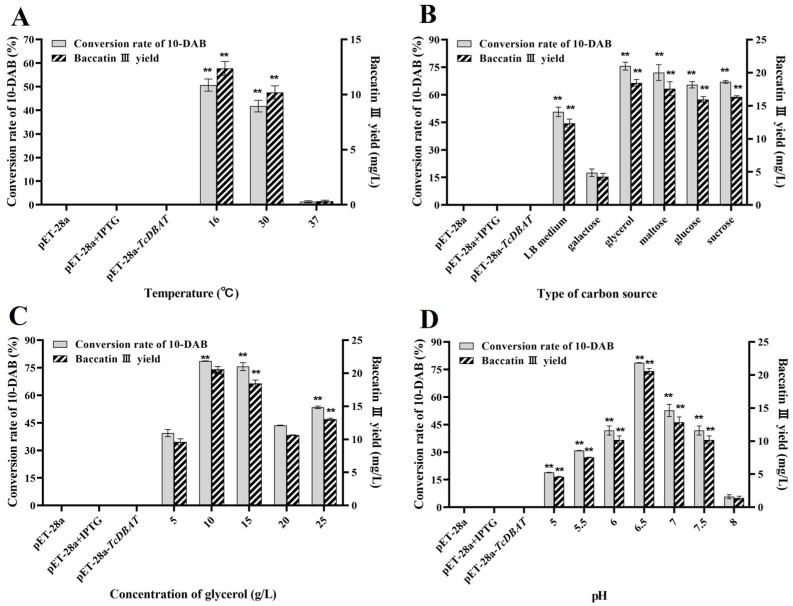
Process establishment for the whole-cell catalytic synthesis of baccatin III based on needles of *Taxus chinensis*. (**A**) Optimization of temperature on the synthesis of baccatin III. (**B**) Optimization of carbon source type on the synthesis of baccatin III. (**C**) Optimization of carbon source concentration on the synthesis of baccatin III. (**D**) Optimization of pH on the synthesis of baccatin III. ** represents significant differences at the *p* < 0.01 level.

Figure 4 shows the optimization for temperature, carbon source type, carbon source concentration and pH with a view to establish an integrated whole-cell catalytic process for the production of baccatin III. The yields of baccatin III shown here are all the yields after subtracting the presence of *Taxus* needles. A high temperature was also not beneficial for the biotransformation of 10-DAB in *Taxus* needles (Figure 4A). 16 °C was determined to be the optimal catalytic reaction temperature and the yield of baccatin III reached 12.36 mg/L, which was 30% of that with single 10-DAB substrate. Generally, a high temperature within a certain range promotes mass transfer and the diffusion of secondary metabolites from plant cells to liquid states. This result indicated that a high temperature may increase the extraction rate of 10-DAB from needles but is not conducive to further acetylation. This is primarily determined by the thermal sensitivity of TcDBAT, and may also be related to the diverse metabolite composition and the complex structure of plant cells [32,33,34]. Although the catalytic efficiency needs to be further improved, this result can prove that the integration of the extraction of 10-DAB and the biotransformation synthesis of baccatin III can be achieved by directly using *Taxus* needles as a substrate.

Different types and concentrations of carbon sources have a significant effect on the yield of baccatin III by influencing the supply of acetyl-CoA. When glycerol is used as the carbon source, the yield of baccatin III is the highest at 18.45 mg/L, which is consistent with the optimal type of carbon source of a single 10-DAB substrate (Figure 4B). In addition, the difference in the yield of baccatin III between other carbon sources and glycerol was reduced. This may because the endogenous metabolic process in plant cells involves the synthesis and consumption of acetyl-CoA, which increases the content of acetyl-CoA on the whole and weakens the dependence of the conversion from carbon source to acetyl-CoA. The conversion rate of 10-DAB and yield of baccatin III are highest at a glycerol concentration of 10 g/L (Figure 4C), which was lower than the optimal concentration of single 10-DAB substrate (15 g/L). This also indicates that the demand for acetyl-CoA from carbon sources is reduced in the target acetylation process. This may because plant metabolism produces a certain amount of acetyl-CoA to provide partial acetyl donors for the biotransformation of 10-DAB.

The target whole-cell catalytic process has been found to occur more readily in a slightly acidic condition (pH = 6.5) when single 10-DAB substrate was used. Consistent results were shown when needles was applied (Figure 4D). The conversion of 10-DAB and the yield of baccatin III gradually increased and then decreased with the gradual increase in initial pH and reached a maximum at a pH of 6.5 (20.58 mg/L). This indicated that both over-acidic and over-basic conditions were unfavorable for baccatin III production. A low initial pH may inhibit the growth of the engineered strain, leading to a decrease in total enzyme activity and baccatin III accumulation. A higher pH promoted the reversible reaction of baccatin III to 10-DAB. So, a pH of 6.5 was determined as the optimal catalytic condition.

### 2.6. Production of Baccatin III from Taxus Needles under Optimal Conditions

**Figure 5:**
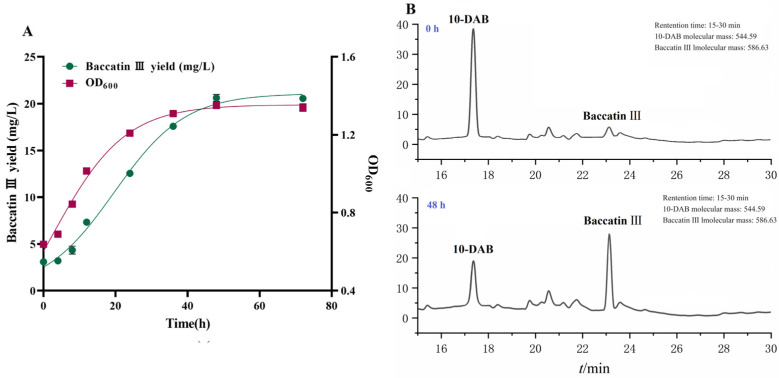
Kinetic study of the whole-cell catalytic synthesis of baccatin III based on needles of *Taxus chinensis*. (**A**) Experimental values and fitted curves of engineered bacteria growth and baccatin III production during whole-cell catalytic fermentation. (**B**) Baccatin III production by recombinant *TcDBAT* biotransformation of *Taxus chinensis* needles for 48 h.

The kinetic curve of baccatin III production from fermented *Taxus chinensis* needles can reflect the dynamic pattern of baccatin III accumulation and provide a reference for the determination of the reaction termination time in the production process. The experimental values of baccatin III concentration, bacterial concentration and the fitted curves of the nonlinear (Logistic) model under the optimal whole-cell catalysis conditions (16 °C, LB + 10 g/L glycerol, pH = 6.5) are shown in Figure 5A. The R^2^ value of the two models exceeded 0.99, indicating that the models were basically consistent with the experimental results and could be used to describe the dynamic changes of the baccatin III content and bacterial concentration. The concentration of bacteria (OD_600_) remained at a low level within 4 h, indicating that the bacteria were in the adaptation period. The engineered bacteria multiplied rapidly with the extension of fermentation time and the concentration increased rapidly. This indicated that the strain entered the logarithmic growth phase, and the expression of TcDBAT and the content of acetyl-CoA increased continuously. The concentration of bacterial did not increase after 48 h and remained in a stable range.

The variation curve of the baccatin III concentration in the fermentation broth was similar to the growth of engineered bacteria but with a time lag. The baccatin III concentration also increased slowly within 8 h. Then also increased rapidly when the bacteria entered the logarithmic growth stage. The target substrate 10-DAB was gradually extracted from the needles in above two stages. When the engineered bacteria entered the stable phase, the concentration of baccatin III was also maintained in a relatively stable range without significant increase. Overall, there was a positive correlation between the variation in baccatin III concentration and bacterial concentration during the biotransformation process. The whole-cell transformation process may belong to the growth coupling type. Therefore, 48 h can be identified as the optimum termination time for the production of baccatin III based on *Taxus chinensis* needles (Figure 5B). In conclusion, the efficient production process of baccatin III from the whole-cell biotransformation of *Taxus chinensis* needles was established, which was 16 °C, LB + 10 g/L glycerol, pH = 6.5, and 48 h. This process can realize the efficient bioconversion of 40 g/L needles into 20.66 mg/L baccatin III, which is 67.15% higher than the yield before optimization.

## 3. Discussion

In this study, the *TcDBAT* was integrated into *E. coli* and a whole cell catalytic process for the efficient synthesis of baccatin III based on *Taxus* needles was established. The comparison with the published baccatin III production efficiencies is necessary to evaluate the application prospects of this study (Table 1). Wang et al. constructed a hybrid metabolic pathway of acetyl-CoA and DBAT to produce baccatin III. The recombinant strain was cultured in TB medium and the yield of baccatin III reached 0.52 g/L after fermentation with 10-DAB for 48 h [31]. This result is the highest efficiency for the synthesis of baccatin III using biotransformation at present. However, it was achieved using fed-batch high density fermentation in a 3 L bioreactor, which was different from the fermentation method in this study. Therefore, the subsequent comparison was directed toward whole-cell catalysis using the shake flask fermentation, which is consistent with the present study. Huang et al. screened a variety of low-cost compounds such as N-acetyl-D-glucosamine to replace acetyl-CoA to provide acetyl groups for the target acetylation process, but the yield of baccatin III was only 1.49 mg/L [35]. This result suggested that acetyl-CoA is the best choice of acetyl donor for the bioconversion of 10-DAB to baccatin III. However, the cost of applying acetyl-CoA to baccatin III production is high. Therefore, Huang et al. increased the yield of baccatin III from 8.60 mg/L to 28.80 mg/L by adding additional carbon source to the TB medium [36]. Their results showed that increasing the supply of carbon source could produce excess acetyl-coA to provide acetyl group while ensuring the growth of strains. Therefore, glycerol was added to LB medium and pH was adjusted to a slightly acidic level in this study according to the influence of carbon source and pH on the whole cell catalytic process. The yield of baccatin III can reach 66.40 mg/L, which is higher than the current maximum yield using the shaker fermentation.

In addition, almost all studies using engineered bacteria to produce baccatin III have used 10-DAB as a substrate for the biotransformation. However, the main source of 10-DAB is extracted and isolated from *Taxus*, and the production process is complicated. Therefore, a novel synthesis strategy for baccatin III was provided by integrated extraction of 10-DAB and in situ whole-cell catalysis with *Taxus* needles as substrate directly. The engineered strain converted 2 g needles (about 1.22 mg 10-DAB) to 20.66 mg/L baccatin III within 48 h under optimized catalytic conditions. The conversion rate reached 78.60%, exceeding the highest reported conversion rate (61.36%). Although the yield of baccatin III was lower compared to the bioconversion with 10-DAB as substrate, it could still reach 71.74% of the reported maximum yield (28.80 mg/L). The above results indicated that using the in situ whole-cell catalysis of *Taxus* needles to synthesize baccatin III with genetic engineered bacteria is a promising biotransformation strategy.

## 4. Materials and Methods

### 4.1. Materials

Total RNA of *Taxus chinensis* was prepared using a universal rapid extraction kit for total plant RNA from BioTeke Corporation Co., Ltd. (Bejing, China). MMLV Reverse Transcriptase, PrimeSTAR^®^ Max DNA Polymerase, restriction enzymes, T4 DNA ligase and DNA Marker were purchased from TAKARA Co., Ltd. (Dalian, China). *E. coli* strains Rosetta (DE3) and plasmids pET-28a(+) were used for heterologous protein expression.

10-DAB and baccatin III standards (purity ≥ 98%) were purchased from Weikeqi biotech Co., Ltd. (Sichuan, China). Isopropyl β-D-thiogalactopyranoside (IPTG) was purchased from MACKLIN Co., Ltd. (Shanghai, China). Kanamycin sulfate was purchased from Solarbio Co., Ltd. (Bejing, China). All other chemicals are analytical grade unless otherwise indicated.

### 4.2. Plasmid Construction

The 10-deacetylbaccatin Ⅲ acetyltransferase gene (*TcDBAT*) was amplified from *Taxus chinensis* cDNA after extraction for total RNA and reverse transcription. The primers used for the constructing of plasmid is shown in Table S1. *TcDBAT* was inserted into pET-28a(+) using restriction sites *BamH*Ⅰ/*Xho*Ⅰ to construct pET-28a-*TcDBAT*. Then it was verified using gene sequencing and sequence alignment.

### 4.3. Heterologous Expression of TcDBAT

The engineering bacteria was inoculated in a 5 mL LB medium and shake-flask cultured at 37 °C. Then 1 mL fermentation broth was inoculated into 49 mL LB medium in which the final concentration of kanamycin was 50 μg/mL. IPTG was added when OD reached the specified value of 600 nm. In order to minimize the formation of inclusion body, the culture was induced under 16 °C for 4 h. Two factors (induction timing and induction concentration) during the process of induction expression in *E. coli* were investigated. Gradient conditions with a cell density of OD_600_ (0.4, 0.6, 0.8 and 1.0) and IPTG concentration (0.02, 0.06, 0.1 and 0.14 mM) were set, respectively. The culture broth was harvested after 4 h of IPTG induction and used for TcDBAT expression assays. The homogenate was centrifuged at 8000 rpm for 5 min, and the precipitate was resuspended in a phosphate buffer solution with a pH of 7.5 (25 mM Na_2_HPO_4_ and 25 mM KH_2_PO_4_). The resuspension cells were disrupted using ultrasonication for 8 min to prepare the cell homogenate. The homogenate was centrifuged at 12,000 rpm for 40 min, the supernatant and the cellular debris were detected using sodium dodecyl sulfate-polyacrylamide gel electrophoresis (SDS-PAGE).

### 4.4. The Effect of Different Factors on the Biotransformation of 10-DAB

In order to investigate the factors influencing the whole-cell catalytic process, 2 mg/mL of 10-DAB standard solution was prepared as the reaction substrate. Then, 2 mL of a 10-DAB standard solution and IPTG were directly added to the 50 mL fermentation broth with the engineered bacteria. Afterwards, the influence pattern for the factors in the acetylation process was explored, including temperature, carbon source type, carbon source concentration, and pH. 3 mL catalytic reaction solution was collected after 48 h.

### 4.5. Production of Baccatin Ⅲ by Biotransformation Based on Taxus Needles

In order to confirm the feasibility of applying the whole-cell catalysis of engineered bacteria directly to the biotransformation of *Taxus* needles and determine the optimal process conditions, *Taxus* needles need to be prepared as the reaction substrate. Mature needles of four-year-old *Taxus chinensis* were collected and ground with liquid nitrogen. Considering that the volume of plant tissue would affect the fermentation process, 2 g needles were added into 50 mL of fermentation broth with the engineered bacterium. The factors in the acetylation process, including temperature, carbon source type, carbon source concentration and pH were optimized using ground *Taxus* needles as substrates. The optimal catalytic conditions were determined after 48 h under the indication of conversion rate of 10-DAB and baccatin III yield.

In order to calculate the conversion of 10-DAB and the yield of baccatin III in whole-cell catalysis process, the contents of 10-DAB and baccatin III released from the needles of *Taxus* during fermentation should be determined as the content before reaction. Therefore, 2 g of milled needles were added to the same volume of fermentation broth of engineered bacteria without IPTG, and the contents of 10-DAB and baccatin III in 50 mL fermentation broth were determined using high performance liquid chromatography (HPLC) after 48 h, which were 1.22 mg and 0.152 mg, respectively. The yield of baccatin III was calculated by subtracting 3.04 mg/L of baccatin III from the needles of *Taxus*.

### 4.6. High Performance Liquid Chromatography Analytical

The fermentation broth after the catalytic reaction was centrifuged at 12,000 rpm for 10 min, the supernatant was harvested and the solvent was removed. Then the concentration of baccatin III and 10-DAB after whole-cell catalysis were analyzed using HPLC after the addition of 200 μL of chromatographic methanol. All assays were carried out in triplicate.

The concentrations of two target compounds were determined using HPLC system from Dalian Elite Analytical Instruments Co., Ltd. (Dalian, China), which was equipped with a Supersil PFP column (250 mm × 4.6 mm, 5 µm) and an ultraviolet (UV) detector. The detection wavelength of both compounds was 227 nm and the flow rate was 1 mL/min. The mobile phase consisted of solvent A (methanol) and solvent B (water). The gradients were as follows: 0–5 min, 15–50% A; 5–7 min, 50–50% A; 7–15 min, 50–60% A; 15–20 min, 60–65% A; 20–23 min, 65–65% A; 23–31 min, 65–80% A; 31–41 min, 80–90% A; 41–45 min, and 90–90% A. The contents of 10-DAB and baccatin III (mg) were determined using the linear regression equation (Figure S2). The bioconversion rate of 10-DAB and yield of baccatin III were calculated using formula (1) and (2), respectively.

Bioconversion rate of 10-DAB (%) = (W_1_ − W_2_)/W_1_ × 100% (1)

Yield of baccatin III = W_3_ − W_4_ (2)

where W_1_ and W_2_ are the content of 10-DAB before the reaction (2 mg or 1.22 mg) and after the reaction. W_3_ and W_4_ are the content of baccatin III after the reaction and before the reaction (0 or 0.152 mg).

### 4.7. Statistic Analysis

The bacterial concentration and baccatin III concentration in the fermentation broth during biotransformation process were measured and nonlinear fitting was performed by Logistic kinetic model. The one-way ANOVA with Tukey’s post hoc test was used in GraphPad Prism 9.0 software to calculate the statistical significance of differences. The difference was considered to be significant when *p* < 0.05 and expressed as “*”. An extremely significant difference was expressed with “**” when *p* < 0.01.

## 5. Conclusions

In this study, a novel production process of baccatin Ⅲ based on *Taxus* needles was established by using *E. coli* expressing *TcDBAT*. The promotion effects of the glycerol supply and the slightly acidic conditions on the biotransformation efficiency were clarified using 10-DAB as a substrate. The in situ whole-cell catalytic process was optimized and established to convert 40 g/L of *Taxus chinensis* needles into 20.66 mg/L baccatin III after extraction. The great potential of applying the *Taxus* needle resources to baccatin III production has been demonstrated. These results provide an alternative strategy for the efficient production of baccatin III using whole cell catalysis and the improvement of the resource utilization rate of *Taxus* needles.

## Figures and Tables

**Table 1:** Comparison with the yield of baccatin III produced by biotransformation.

Conversion Rate of 10-DAB	Baccatin Ⅲ Yield	Substrate	Condition	References
89.84%	0.52 g/L	544.59 mg/L 10-DAB	TB; 30 °C; pH = 5.8; 48 h	[31]
3.17%	1.49 mg/L	43.57 mg/L 10-DAB + 1M N-acetyl-D-glucosamine	TB; 20 °C; pH = 7.4; 36 h	[35]
61.36%	28.80 mg/L	43.57 mg/L 10-DAB	TB + 10g/L glycerol; 25 °C; pH = 7.0; 48 h	[36]
18.33%	8.60 mg/L	43.57 mg/L 10-DAB	TB; 20 °C; pH = 7.0; 48 h	[36]
77.05%	66.40 mg/L	80 mg/L 10-DAB	LB + 15 g/L glycerol; 16 °C; pH = 6.5; 48 h	This study
78.60%	20.66 mg/L	40 g/L needles (24.40 mg/L 10-DAB)	LB + 10 g/L glycerol; 16 °C; pH = 6.5; 48 h	This study

## Data Availability

The original data of this present study are available from the corresponding authors.

## Supplementary Materials

The following supporting information can be downloaded at: https://www.mdpi.com/article/10.3390/molecules29112586/s1, Figure S1: Optimization of recombinant TcDBAT induced expression conditions. (A) Induction timing. (B) Inducer concentration. Figure S2: HPLC calibration curves of 10-DAB (A) and baccatin Ⅲ (B). Table S1: Primers used in this study.

## References

[1] Wang F., Huang Q., Su H., Sun M., Wang Z., Chen Z., Cui H. (2023). Self-assembling paclitaxel-mediated stimulation of tumor-associated macrophages for postoperative treatment of glioblastoma. Proc. Natl. Acad. Sci. USA.

[2] He W., Leng X., Mao T., Luo X., Zhou L., Yan J., Han Y. (2022). Toripalimab plus paclitaxel and carboplatin as neoadjuvant therapy in locally advanced resectable esophageal squamous cell carcinoma. Oncologist.

[3] Zhang Y., Chen H., Mo H., Hu X., Gao R., Zhao Y., Liu Z. (2021). Single-cell analyses reveal key immune cell subsets associated with response to PD-L1 blockade in triple-negative breast cancer. Cancer Cell.

[4] Fukaya K., Tanaka Y., Sato A.C., Kodama K., Yamazaki H., Ishimoto T., Chida N. (2015). Synthesis of Paclitaxel. 1. Synthesis of the ABC ring of Paclitaxel by SmI2-mediated cyclization. Org. Lett..

[5] Zhang S., Ye T., Liu Y., Hou G., Wang Q., Zhao F., Meng Q. (2023). Research advances in clinical applications, anticancer mechanism, total chemical synthesis, semi-synthesis and biosynthesis of paclitaxel. Molecules.

[6] Arnone A., Bava A., Fronza G., Malpezzi L., Nasini G. (2010). Microbial transformations of the taxan ring of 10-DAB by some strains of the fungus *Curvularia lunata*: Formation of the bis-abeotaxanes wallifoliol and 4-deacylwallifoliol. J. Nat. Prod..

[7] Yanagi M., Ninomiya R., Ueda Y., Furuta T., Yamada T., Sunazuka T., Kawabata T. (2016). Organocatalytic site-selective acylation of 10-deacetylbaccatin III. Chem. Pharm. Bull..

[8] Holton R.A., Zhang Z., Clarke P.A., Nadizadeh H., Procter D.J. (1998). Selective protection of the C(7) and C(10) hydroxyl groups in 10-deacetyl baccatin III. Tetrahedron Lett..

[9] You L.F., Huang J.J., Lin S.L., Wei T., Zheng Q.W., Jiang B.H., Guo L.Q. (2019). In vitro enzymatic synthesis of baccatin III with novel and cheap acetyl donors by the recombinant taxoid 10β-O-acetyl transferase. Biocatal. Biotransformation.

[10] Loncaric C., Merriweather E., Walker K.D. (2006). Profiling a taxol pathway 10β-acetyltransferase: Assessment of the specificity and the production of baccatin III by in vivo acetylation in *E. coli*. Chem. Biol..

[11] You L.F., Wei T., Zheng Q.W., Lin J.F., Guo L.Q., Jiang B.H., Huang J.J. (2018). Activity essential residue analysis of Taxoid 10β-O-acetyl transferase for enzymatic synthesis of baccatin. Appl. Biochem. Biotechnol..

[12] Xiong X., Gou J., Liao Q., Li Y., Zhou Q., Bi G., Yan J. (2021). The *Taxus* genome provides insights into paclitaxel biosynthesis. Nat. Plants.

[13] Salehi M., Farhadi S., Moieni A., Safaie N., Hesami M. (2021). A hybrid model based on general regression neural network and fruit fly optimization algorithm for forecasting and optimizing paclitaxel biosynthesis in *Corylus avellana* cell culture. Plant Methods.

[14] Adegoke T.V., Yang B., Xing F., Tian X., Wang G., Tai B., Jahan I. (2022). Microbial enzymes involved in the biotransformation of major mycotoxins. J. Agric. Food Chem..

[15] Zhao H., Jiao W., Xiu Y., Zhou K., Zhong P., Wang N., Yu S. (2022). Enzymatic biotransformation of gypenoside XLIX into gylongiposide I and their antiviral roles against enterovirus 71 in vitro. Molecules.

[16] Yang S.C., Ting W.W., Ng I.S. (2022). Effective whole cell biotransformation of arginine to a four-carbon diamine putrescine using engineered *Escherichia coli*. Biochem. Eng. J..

[17] Mao X., Qian X., Lin J., Wei D. (2023). Engineering gluconobacter oxydans for efficient production of 3,4-dihydroxybutunate or 1,2,4-butanetriol from D-xylose. Biochem. Eng. J..

[18] Han F., Kang L.Z., Zeng X.L., Ye Z.W., Guo L.Q., Lin J.F. (2014). Bioproduction of baccatin III, an advanced precursor of paclitaxol, with transgenic *Flammulina velutipes* expressing the 10-deacetylbaccatin III-10-O-acetyl transferase gene. J. Sci. Food Agric..

[19] Jiang S., Zhang Y., Zu Y., Wang Z., Fu Y. (2010). Antitumor activities of extracts and compounds from water decoctions of *Taxus cuspidate*. Am. J. Chin. Med..

[20] Kajani A.A., Bordbar A.K., Esfahani S.H.Z., Khosropour A.R., Razmjou A. (2014). Green synthesis of anisotropic silver nanoparticles with potent anticancer activity using *Taxus baccata* extract. Rsc Adv..

[21] Wianowska D., Hajnos M.Ł., Dawidowicz A.L., Oniszczuk A., Waksmundzka-Hajnos M., Głowniak K. (2009). Extraction methods of 10-deacetylbaccatin III, paclitaxel, and cephalomannine from *Taxus baccata* L. twigs: A comparison. J. Liq. Chromatogr. Relat. Technol..

[22] Yu J., Wang Y., Qian H., Zhao Y., Liu B., Fu C. (2012). Polyprenols from the needles of *Taxus chinensis* var. mairei. Fitoterapia.

[23] Shi Q.W., Sauriol F., Mamer O., Zamir L.O. (2003). New minor taxane derivatives from the needles of *Taxus canadensis*. J. Nat. Prod..

[24] Meng A.P., Li J., Pu S.B. (2018). Chemical constituents of leaves of *Taxus chinensis*. Chem. Nat. Compd..

[25] Zhao Y., Wang F.S., Peng L.Y., Li X.L., Xu G., Luo X.X., Zhao Q.S. (2006). Taxoids from *Taxus chinensis*. J. Nat. Prod..

[26] Sadykhov E.G., Serov A.E., Voinova N.S., Uglanova S.V., Petrov A.S., Alekseeva A.A., Tishkov V.I. (2006). A comparative study of the thermal stability of formate dehydrogenases from microorganisms and plants. Appl. Biochem. Microbiol..

[27] Loughran N.B., O’Connell M.J., O’Connor B., Ó’Fágáin C. (2014). Stability properties of an ancient plant peroxidase. Biochimie.

[28] Liu W., Zhang B., Jiang R. (2017). Improving acetyl-CoA biosynthesis in *Saccharomyces cerevisiae* via the overexpression of pantothenate kinase and PDH bypass. Biotechnol. Biofuels.

[29] Guo L., Lu J., Gao C., Zhang L., Liu L., Chen X. (2021). Dynamic control of the distribution of carbon flux between cell growth and butyrate biosynthesis in *Escherichia coli*. Appl. Microbiol. Biotechnol..

[30] Zhang S., Yang W., Chen H., Liu B., Lin B., Tao Y. (2019). Metabolic engineering for efficient supply of acetyl-CoA from different carbon sources in *Escherichia coli*. Microb. Cell Factories.

[31] Wang H., Zhang B.Y., Gong T., Chen T.J., Chen J.J., Yang J.L., Zhu P. (2021). Construction of acetyl-CoA and DBAT hybrid metabolic pathway for acetylation of 10-deacetylbaccatin III to baccatin III. Acta Pharm. Sin. B.

[32] Yu S.H., Ni Z.Y., Zhang J., Dong M., Sauriol F., Huo C.H., Cong B. (2009). Taxusecone, a novel taxane with an unprecedented 11, 12-secotaxane skeleton, from *Taxus cuspidata* needles. Biosci. Biotechnol. Biochem..

[33] Sims D.A., Gamon J.A. (2002). Relationships between leaf pigment content and spectral reflectance across a wide range of species, leaf structures and developmental stages. Remote Sens. Environ..

[34] Kintsurashvili L.G. (2013). Diterpene alkaloid karacoline from *Taxus baccata* growing in Georgia. Chem. Nat. Compd..

[35] Huang J.J., Wei T., Lin J.F., Guo L.Q., Han W.F., Han P.Y., Ye A.Q. (2020). High-effective biosynthesis of baccatin Ⅲ by using the alternative acetyl substrate, N-acetyl-d-glucosamine. J. Appl. Microbiol..

[36] Huang J.J., Wei T., Ye Z.W., Zheng Q.W., Jiang B.H., Han W.F., Lin J.F. (2022). Microbial cell factory of baccatin III preparation in *Escherichia coli* by increasing DBAT thermostability and in vivo acetyl-CoA supply. Front. Microbiol..

