# Supplementary material for: An Integrated Strategy Based on 10-DAB Extraction and In Situ Whole-Cell Biotransformation of Renewable Taxus Needles to Produce Baccatin III
Source: Molecules. 2024 May 31;29(11):2586. doi: 10.3390/molecules29112586 (PMC11173793; doi:10.3390/molecules29112586)
Supplement: Supplementary file 1 [file molecules-29-02586-s001.zip › molecules-3017960-supplementary.pdf]

Supplementary data to:

An integrated strategy based on 10-DAB extraction and in situ whole-cell biotransformation of renewable *Taxus* needles to produce baccatin III

Ping Kou, Yingying Yu, He Wang, Yuchi Zhang, Zhaoxia Jin, Fang Yu *

School of Biological Engineering, Dalian Polytechnic University, Dalian 116034, People's Republic of China

* Corresponding author: yufang@dlpu.edu.cn.

Table S1 Primers used in this study

Primers	Sequences (5'-3')
*Bam*HI-*TcDBAT*-F	GCGGATCCATGGCAGGCTCAACAGAAT
*Xho*I-*TcDBAT*-R	GCGGATCCATGGCAGGCTCAACAGAAT

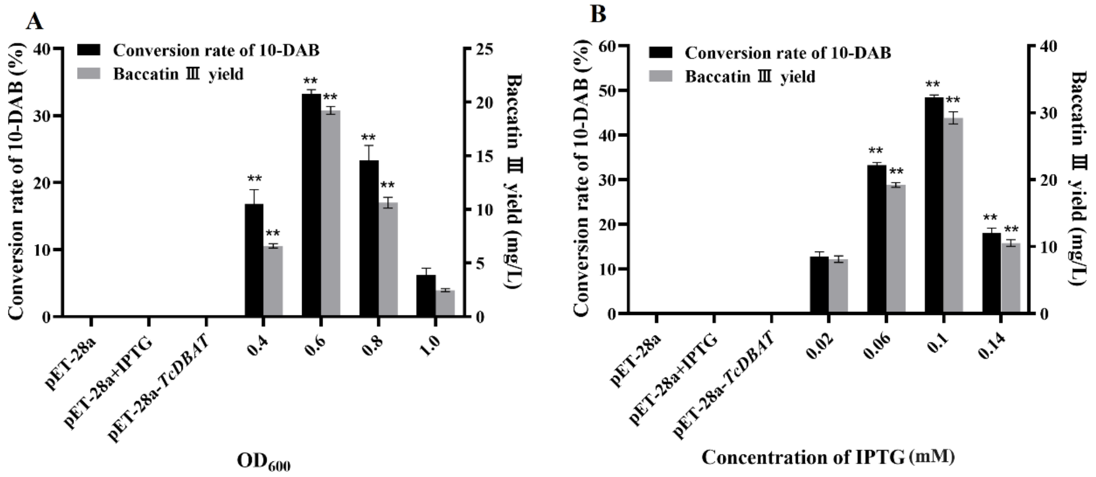

Figure S1. Optimization of recombinant TcDBAT induced expression conditions. (A) Induction timing. (B) Inducer concentration.

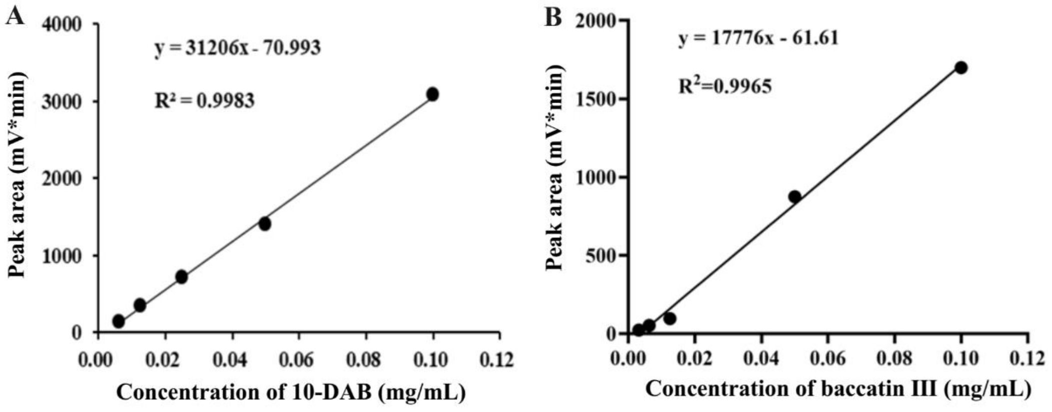

Figure S2. HPLC calibration curves of 10-DAB (A) and baccatin III (B).
